# Therapeutic application of a jumbo bacteriophage against metallo-β-lactamase producing *Pseudomonas aeruginosa* clinical isolates

**DOI:** 10.1186/s12929-025-01169-z

**Published:** 2025-08-11

**Authors:** Paschalis Paranos, Dimitrios Skliros, Nikita Zrelovs, Panagiota-Christina Georgiou, Karina Svanberga, Andris Kazaks, Marios Kostakis, Nikolaos Thomaidis, Emmanouil Flemetakis, Joseph Meletiadis

**Affiliations:** 1 Clinical Microbiology Laboratory, Medical School, Attikon University Hospital, National and KapodistrianUniversity of Athens, Rimini 1, Haidari, Athens, Greece; 2 Laboratory of Environmental Biotechnology, Department of Biotechnology, School of Applied Biology and Biotechnology, Agricultural University of Athens, Athens, Greece; 3 Latvian Biomedical Research and Study Centre, Riga, Latvia; 4 Laboratory of Analytical Chemistry, Department of Chemistry, National and Kapodistrian University of Athens, Athens, Greece

**Keywords:** Jumbo phages, Carbapenemase producing *P. aeruginosa*, Metallo β-lactamases, Lytic activity

## Abstract

**Background:**

Therapeutic options against metallo-β-lactamase producing *P. aeruginosa* (MBL-PA) are limited due to multi-drug resistance. A jumbo phage isolated from wastewater in Greece was characterized microbiologically and genetically and evaluated for its potential as a therapeutic agent alone or in combination with antibiotics in an experimental thigh infection mouse model.

**Methods:**

The host range of the jumbo phage vB_PaerM_AttikonH10 (AttikonH10) against 20 MBL-PA clinical isolates and 10 susceptible strains, one-step phage growth and growth curves of mid-exponential phase bacteria upon addition of the phage were analyzed. Whole-genome sequencing was performed and the de novo assembled complete phage genome was compared with other jumbo phages. In vivo pharmacokinetics in different tissues as well as the efficacy of two dosing regimens 10^9^ and 10^6^ PFU/mouse administered intraperitoneally alone and in combination with amikacin (384 mg/kg/day) was tested against an MBL-PA clinical isolate in murine thigh infection model.

**Results:**

The phage formed small plaques in double-layer agar and demonstrated clear or semi-clear lysis in 83.3% (25/30) of *P. aeruginosa* clinical isolates. Growth curves showed a > 94% growth inhibition in the presence of phage even at the lowest multiplicity of infection ratio tested (10^–5^). Whole genome analysis indicated a jumbo dsDNA phage with 278,406 bp (36.92% GC) belonging to *Phikzvirus* that is predicted to host up to 413 putative ORFs and 6 tRNA genes. No known lysogeny-associated genes, virulence factors, or antimicrobial resistance genes were identified within the genome. Phage titres 10^4^–10^6^ PFU/tissue were detected in all mouse tissues with elimination half-life of 3.4 h except in bronchoalveolar lavage where no phages were found. Only the high phage dose (10^9^ PFU/mouse) reduced bacterial load in thigh by 1.09 log_10_ cfu/thigh compared to placebo, similar to amikacin monotherapy (1.19 log_10_ cfu/thigh), while their combination achieved a greater reduction of 2.07 log_10_ cfu/thigh compared to each monotherapy (p = 0.0044–0.0048).

**Conclusions:**

The newly reported *Phikzvirus* jumbo phage AttikonH10 demonstrated a broad host range, strong lytic activity and synergistic effects with amikacin against MBL-PA isolates making it a candidate for phage therapy.

**Supplementary Information:**

The online version contains supplementary material available at 10.1186/s12929-025-01169-z.

## Background

*Pseudomonas aeruginosa* commonly causes life-threatening nosocomial infections in critically ill and immunocompromised patients [1]. World Health Organization (WHO) in 2024 has classified *P. aeruginosa* as a “high priority pathogen”, highlighting the continuous need for innovative approaches to address the impact of carbapenem-resistant *P. aeruginosa* (CRPA) on health care, due to high fatal burden among immunocompromised individuals [2]. The increasing presence of CRPA isolates in healthcare settings has become a serious public health threat. The threat that these bacteria may pose to the public is perfectly illustrated by the current international, prospective cohort study demonstrating that carbapenemase-producing CRPA isolates were associated with higher mortality rates compared to non-carbapenemase-producing CRPA [3]. Apart from the USA, 30–69% of CRPA isolates from other regions around the world (Asia, Australia and Singapore, Middle East, South and Central America) had a carbapenemase with subsequently higher levels of resistance to meropenem and other antipseudomonal drugs [3]. Among the CRPA isolates, those that are producing metallo-β-lactamases (MBLs) are of major concern, because most antibiotics used against *P. aeruginosa*, including the more recent antipseudomonal drugs (e.g., ceftolozane-tazobactam, ceftazidime-avibactam, meropenem-vaborbactam) are inactive [4].

Bacteriophages (phages) are among the most abundant and diverse entities on the planet. Based on “Hendrix product” it is estimated that total number of virus-like particles on Earth remains close to 10^31^, outnumbering microbial cells in most natural environments [5]. However, the genetic diversity of bacteria like *P. aeruginosa* and generally narrow host specificity of known bacteriophages pose a challenge towards the discovery of novel phages that would demonstrate potent lytic activity, wide host range, resistance to bacterial host defense mechanisms, adequate tissue distributions and in vivo activity for use in medicinal applications [6]. Jumbo phages (those with genomes > 200 kb), exemplified by phiKZ-like phages, exhibit numerous intriguing characteristics, including pan-resistance to recognized DNA-targeting immune systems [7]. Commercial phage therapeutic polyvalent mixtures were previously shown to contain various phiKZ-like phages as permanent components [8]. The capacity of such phages to overcome a range of host cell defences and deliver high final yields after a chain of productive infections in a susceptible host population is one of its appealing qualities when utilized in commercial preparations [8, 9].

The majority of the so far isolated jumbo phages infect Gram-negative hosts [9]. Currently, more than 500 different jumbo phage complete genomes are publicly available in GenBank, and 79 of them have *Pseudomonas* indicated as a host. Over the last two years, studies have identified five new phiKZ-like jumbo phages that exhibit lytic activity against clinical *P. aeruginosa* strains [10–12], but efficacy data against MBL-producing clinical isolates are absent. These findings highlight the emerging potential of jumbo phages as therapeutic candidates against CRPA. A thorough biological and genomic characterization of phages is of great significance to evaluate potential use in phage therapy. Characteristics such as lytic activity, host range, burst size and absence of any known potentially harmful genes, such as those encoding the proteins associated with the temperate lifecycle (e.g., integrase), antimicrobial resistance, and host virulence modifiers [13] should be assessed for any phage candidate for phage therapy. Furthermore, in vivo pharmacokinetics of jumbo phages have not been studied. This is very important for evaluation of their therapeutic application as it has been previously found that phage morphology may have an impact on its pharmacokinetics since smaller sized bacteriophages show increased rates of intracellular uptake in vitro [14], large bacteriophage virions are easier to filter out in vivo than small ones [14], whereas the capacity to overcome serum neutralization can vary from one bacteriophage to another [15]. Finally, phages are usually administered together with antibiotics. As phages may interact with antibiotics producing either synergistic or antagonistic effects, assessment of phage + antibiotic combination is crucial [16].

In the current study, phenotypic and genomic characterization of a newly isolated *Pseudomonas aeruginosa* jumbo phage vB_PaerM_AttikonH10 (AttikonH10), demonstrating an efficient lytic activity against multiple MBL-producing *P. aeruginosa* clinical isolates, was described. Finally, the PK characteristics of the isolated phage, its distribution in several tissues, as well as its therapeutic efficacy alone, and in combination with antibiotics, was assessed in in vivo experimental thigh infection murine model.

## Methods

### Bacterial strains

A total of 9 non-repetitive well characterized MDR *P. aeruginosa* isolates with distinct resistance mechanisms [1 New Delhi metallo-β-lactamase (NDM)_,_ 3 Verona integron-encoded metallo-β-lactamase (VIM), 1 AmpC_,_ 1 *Klebsiella pneumoniae* carbapenemase-2 (KPC-2) and 3 susceptible], including isolates co-resistant to quinolones (59%), aminoglycosides (52%) and 4th generation cephalosporins (59%), were used for phage isolation and propagation. Presumptive MBLs and AmpCs were previously screened via Combined Disk Synergy Test (CDST) [17] using 0.1 M EDTA and multiplex PCR was used in order to confirm the presence of *bla*_AmpC,_* bla*_KPC,_ *bla*_NDM_ and *bla*_VIM_ genes [18]. Isolates were recovered in different Greek tertiary hospitals during 2016–2021 in order to represent the local epidemiology and were part of Attikon University Hospital collection [19]. Bacterial strains were stored at − 80 °C in Trypticase Soy Broth (TSB) supplemented with 20% glycerol until further use. Strains were revived by subculture on MacConkey agar No. 3 (MAC) (Oxoid, UK) plates and incubated at 37 ^o^C for 24 h. A mid-exponential phase bacteria culture was prepared by incubation a bacterial inoculum of 0.5 McFarland in Luria Bertani (LB) medium supplemented with 10 mM MgSO_4_∙7H_2_O and CaCl_2_ (LB +) for 3-5 h in order to reach O.D_600_ 0.3–0.5 corresponding to 10^8^ cfu/mL.

### Isolation, purification and propagation of jumbo-phage

A total of 50 rectal swabs and 10 stool samples from ICU and pediatric patients from a Greek tertiary hospital as well as 50 influent wastewater samples from Psyttaleia (Athen’s largest sewage treatment plant) were collected during 03/2021–09/2022. Phages were isolated using a typical enrichment protocol [20]. Wastewater samples were pooled every 15 days, while rectal swabs and stool samples were pooled by 10 in 10 ml of 0.9% NaCl. Briefly, 17 ml of unfiltered samples was mixed with 2 ml 10 × LB + and 1 ml of mid-exponential bacterial culture. Falcon containing mixtures were placed in a shaking incubator (orbital shaking, 200 rpm) and incubated at 37 °C for 24 h, in order to stimulate the proliferation of certain phages targeting *P. aeruginosa* isolates that they are exposed to. The day after, 1 ml of each enrichment sample was centrifuged (12,000 g, 10 min, 4 °C) and 10 μl from the supernatant was tested via spot assay using single-layer agar (SLA) against all *P. aeruginosa* isolates, as previously described [21]. Isolated phages were purified through a triple transfer of single plaque formation and propagated until homologous plaques were obtained. Phages were enriched using the highest‐titer where individual plaques were nearly still visible (web pattern), filtered through 0.22 μm syringe filter (Sarstedt, Filtropur S) and titre has been determined using SLA with the corresponding host. Phage stocks were kept in LB broth at 4 °C until further use.

### Determination of host range

In order to determine host range of isolated jumbo phage, 30 clinical *P. aeruginosa* isolates including 10 susceptible to antibiotics and 20 with different resistance mechanisms, 4 NDM and 16 VIM, were tested using the SLA method previously described [22]. Briefly, 100 μl of a strain in the mid-exponential phase were added to 10 ml of 0.5% LB agar (soft agar) and the mixture was poured into petri dishes. After solidification, 10 μl of phage seed was spotted in the bacterial lawn of the SLA plates and after drying, incubated at 37 °C for 24 h. Plates were examined for the presence of plaques and they were assessed based on the clarity of plaques, which was score as transparent (+ + +), semi-transparent (+ +), turbid ( +) and no inhibition (-). In terms of infection, transparent lysis was associated with the killing of the bacterial population, while turbid lysis was associated with phage-resistant bacterial mutants. All the experiments were performed in triplicate on different days.

### One-step phage growth curve

A one-step growth curve was conducted as previously described [23] with slight modifications. Briefly, 10 ml of mid-exponential phase bacterial culture in LB + medium, was infected with the phage at a MOI of 0.01 and incubated at 37 °C. Aliquots were taken every 2 min for the first 15 min to define phage adsorption and then every 10 min for 120 min to define the latent period and burst size. Aliquots were centrifuged (13,000 g × 5 min), the supernatant was serially diluted and 10 μl was spotted onto the host-bacterial lawn using SLA. Phage titers were determined after 24 h of incubation at 37 °C and adsorption rate, adsorption time, latent phase and burst size were calculated. The adsorption rate was calculated by measuring the percentage of virions that attached to the host over a given time interval. Specifically, it was determined using the formula: Adsorption rate (%) = [(N₀ − Nₜ)/N₀] × 100, where *N₀* is the initial phage titer and *Nₜ* is the unadsorbed phage titer found in supernatant after centrifugation at time *t*. The latent phase was defined as the interval between the adsorption of the phages to the bacterial cells and the release of phage progeny [24]. Burst size was calculated as the ratio of the final population of released virions at the end of burst period to the initial count of infected bacterial cells at the end of the latent phase [23]. Three independent replicates were performed.

### Growth curves of host bacteria

Assessment of in vitro effectiveness of the jumbo phage against host bacteria was performed using growth curves. Briefly, the phage at tenfold decreasing MOI ratios ranging from 10 to 0.00001 were mixed with mid-exponential phase bacteria using LB + medium and 200 μl were transferred in a 96-well plate (Nunc 167008, ThermoFischer). The OD_600_ was measured every 30 min for 24 h using spectrophotometer (Infinite M200, Tecan) with incubation at 37 °C and orbital shaking (220 rpm) before each measurement. Four replicates were loaded in every plate for each MOI and growth control, as well as a negative control containing LB medium. Assays were performed in triplicate, in order to explore day-to-day variability. Results for each MOI and each phage were analysed by describing the kinetics using GraphPad Prism 8.0.2. The percentage of growth inhibition was calculated based on OD_600_ reduction after 24 h using the formula: % Growth inhibition = 1 − (OD_MOI_ − OD_0_)/(OD_GC_ − OD_0_) where OD_0_ is the OD at t = 0 h, OD_MOI_ is the OD at t = 24 h at a specific MOI and OD_GC_ is the OD at 24 h for the phage-free growth control. All OD values were background-corrected by subtracting the OD of wells containing only phages. Growth inhibition > 100% indicating that growth reduction was higher than the initial OD at t = 0 h. Moreover, the time required for the bacteria to reach OD of the background at the highest MOI (10), as well as the time needed for the bacteria to regrow post-inoculation of the phage were determined.

### Phage DNA Extraction

DNA extraction was conducted using a Qiagen protocol of the DNeasy Blood and Tissue Kit (Qiagen, Hilden, Germany) with slight modifications [25]. Briefly, having an optimal titer of > 10^10^ PFU/mL, phage particles were concentrated using a standard polyethylene glycol (PEG-8000)/NaCl precipitation. DNase and RNase were used to treat the phage lysate in order to remove free DNA. Phage lysate was mixed with an equal volume of supplied buffer with the addition of 100% EtOH before the first column wash [25]. A yield of at least 10 μg of DNA was successfully retrieved and DNA quality was assessed using Qubit (Thermo Fisher Scientific, Waltham, MA, USA) measurements and agarose gel before library preparation.

### Sequencing and bioinformatic analysis

Approximately 200 ng of dsDNA were fragmented using a Covaris S220 ultrasonicator (Covaris, Woburn, MA, USA) for a desired 550 bp fragment length. Next, TrueSeq DNA nano low-throughput library prep kits (Illumina, San Diego, CA, USA) protocol was followed using an individual adapter #9 from TruSeq DNA single indexes set (Illumina). The quality of the genomic library was verified using an Agilent 2100 bioanalyzer (Agilent, Santa Clara, CA, USA) with a High Sensitivity DNA Kit (Agilent), as well as a Qubit fluorometer (Invitrogen) dsDNA high-sensitivity quantification assay (Invitrogen). Finally, the library was sequenced on the Illumina MiSeq system (Illumina) with the 500-cycle MiSeq reagent kit v2 nano (Illumina, San Diego, CA, USA).

Demultiplexed read datasets were evaluated using FastQC (v0.11.9; [26]) to assess data quality and subsequently processed with the bbduk tool from the bbmap package (v38.69) for quality enhancement in preparation for downstream analyses [27]. Adapter sequences and trailing bases were removed, and reads shorter than 50 bp after trimming were excluded from further analysis. These high-quality reads were then subjected to de novo assembly using Unicycler (v0.4.8; [28]).

Genome annotation was performed using Pharokka (v1.7.1; [29]), employing PHANOTATE [30] for open reading frame (ORF) prediction, ARAGORN [31], and tRNAscan-SE [32] for tRNA gene identification. PHROGs profiles [33] were used for ORF product functional assignments. Antibiotic resistance genes and virulence factors within the predicted phage proteome were identified through comparative analysis against the CARD [34] and VFDB [35] databases, respectively. The functionally annotated genome of the bacteriophage presented in this study is available in the GenBank nucleotide sequence database under accession number PQ741806.1.

To determine relatedness to other bacteriophages completely sequenced up to date and having their genomes publicly available, the nucleotide sequence of phage AttikonH10 was queried against the *Caudoviricetes* sequences (taxid:2731619) via BLASTn [36] webserver with other settings unchanged. All hits to the complete phage genomes were downloaded and subjected to VIRIDIC analysis to calculate the intergenomic distance matrix [37].

For major capsid protein (MCP) and terminase large subunit (TerL) phylogeny reconstruction, the respective amino acid sequences from *Pseudomonas* phage AttikonH10 (XLY82506.1 and XLY82236.1, respectively) were individually queried against proteins from the RefSeq database and the top nine highest-scoring hits were downloaded for context. Multiple sequence alignment and neighbor-joining tree reconstruction (using MSA columns with more than 90% coverage) were performed in MEGA7 [38] assuming uniform rates among sites, with 1000 bootstrap replicates performed as a measure of branch support. The resulting tree was visualized in FigTree (v1.4.4; http://tree.bio.ed.ac.uk/software/figtree/2018).

Complete annotated genomes for the representative isolates of the *Phikzvirus phiKZ* and *Phikzvirus SL2* species (phages phiKZ (NC_004629) and SL2 (NC_042081), respectively), as well as *Pseudomonas* phage KTN4 (KU521356, the closest relative of the studies phage based on the intergenomic similarity) were downloaded, reorganized to ensure collinearity with AttikonH10, and subjected to Clinker (v0.0.23, [39]) analysis to compare their genomic organization and proteomes (using a 70% identity threshold for each protein being compared).

## Thigh infection mouse model

Female CD1 mice, 4–6 weeks old and weighing 22–25 g, obtained from the Pasteur Institute (Athens, Greece), were used for the experiments. Animals were kept in individually ventilated cages with a 10 h/14 h dark/light cycle and had free access (ad libitum) to water and food. Animal experiments were conducted in the unit of animals for medical scientific purposes of ΑΤΤΙΚΟΝ University General Hospital (Athens, Greece) according to EU Directive 2010/63/EU and to the Greek law 2015/2001, which incorporates the Convention for the Protection of Vertebrate Animals used for Experimental and Other Scientific Purposes of the Council of Europe (code of the facility EL 25BIO014, approval no. 1853/2015). All experiments were licensed from the Greek veterinary directorate under the protocol number 1212267/21-10-2024 and animal procedures were performed in strict accordance with the European Health Law of the Federation of Laboratory Animal Science Associations (FELASA). A neutropenic thigh infection model was used. Two doses of cyclophosphamide were administered intraperitoneally (IP) 4 days (150 mg/kg) and 1 day (100 mg/kg) before infection. On the day of infection, the bacterial suspension of an CRPA isolate harboring *bla*_NDM_ gene in the early logarithmic phase was administered intramuscularly at a concentration of 2 × 10^6^ CFU in 50 μL. Phage lysate was filtered through 0.22 μm syringe filter and titre has been determined using SLA with the corresponding host. For endotoxin removal, an organic solvent (1-octanol, Sigma-Aldrich, Athens, Greece) was added to phage lysate at 40% v/v and shaken for 1–3 h at room temperature. The two-phase mixture was then cooled at 4 °C for 1–3 h and separated by centrifugation at 4000 × g for 10 min. The collected aqueous phase was dialyzed using Float-A-Lyzer dialysis tubing (MWCO 1000 kDa) against 25% aqueous ethanol (5 × 4 h), followed by dialysis against 0.15 M sterile NaCl solution (4 × 4 h) [40]. Phage seeds were kept in LB broth at 4 °C until further use.

### In vivo pharmacokinetics

Twenty mice were divided in three groups: the first group (placebo) of 4 mice received 100 μl of 0.9% NaCl via IP route and 2 mice euthanised 1 h after infection and another 2 mice 25 h after infection in order to verify infection and increase of bacterial load in thigh, the second group (high phage dose) of 8 mice received 100 μl (1 × 10^10^ PFU/mL i.e. 10^9^ PFU/mouse), while the third group (low phage dose) of 8 mice received 100 μl (1 × 10^7^ PFU/mL i.e. 10^6^ PFU/mouse) of the purified AttikonH10 via intraperitoneal (IP) route. Two mice from each of the latter two groups were euthanized by cervical dislocation at 1, 4, 8 and 24 h after phage administration. Blood and BAL samples were collected in sterilized tubes by cardiac puncture and by inserting a catheter into the trachea, through a saline solution (1mL) instilled into the bronchioles and aspirated, respectively. Left and right thigh, lungs, liver, spleen, kidneys and brain were collected aseptically from euthanized mice, weighed and homogenized in 2.0 ml 0.9% NaCl aseptically. Blood, BAL and tissue homogenates were diluted further using ten-fold dilutions in 0.9% NaCl. The phage titer was determined by SLA technique as previously described and plotted using GraphPad Prism 8 [21].

### In vivo efficacy of phage alone and in combination with antibiotics

At 1 h post-infection, 100 μl of either 0.9% NaCl (placebo), phage alone at two different dosing regimens (10^9^ and 10^6^ PFU/mouse q12h), amikacin (384 mg/kg/day given as 192 mg/kg q12h), or phage cocktail-amikacin combination were given intraperitoneally to each of the four treatment groups. Combination of AttikonH10 with amikacin was based on findings by Paranos et al*.* [41], who demonstrated in vitro synergistic effects between amikacin and AttikonH10 using checkerboard assays against MBL-producing *P. aeruginosa* clinical isolates. Amikacin dosing regimen has been selected based on previous studies [42] simulating human total exposure after 20 mg/kg/day in patients [43]. At 1 h and 25 h post-infection, the mice were euthanized and the thighs were collected to determine the bacterial load at the site of infection. The collected thighs were homogenized in sterile 0.9% NaCl and ten-fold serial dilutions were cultured on MAC in order to determine the bacterial load in each tissue homogenate and calculate the 24h-log_10_ CFU/thigh change from bacterial load at 1h post-infection before start of treatment. Differences between placebo and monotherapy and between monotherapy and combination therapy were analyzed using one-way ANOVA test followed by Holm-Sidak’s multiple comparison test GraphPad Prism 8 (San Diego, CA).

## Results

### Bacteriophage isolation

**Fig. 1:**
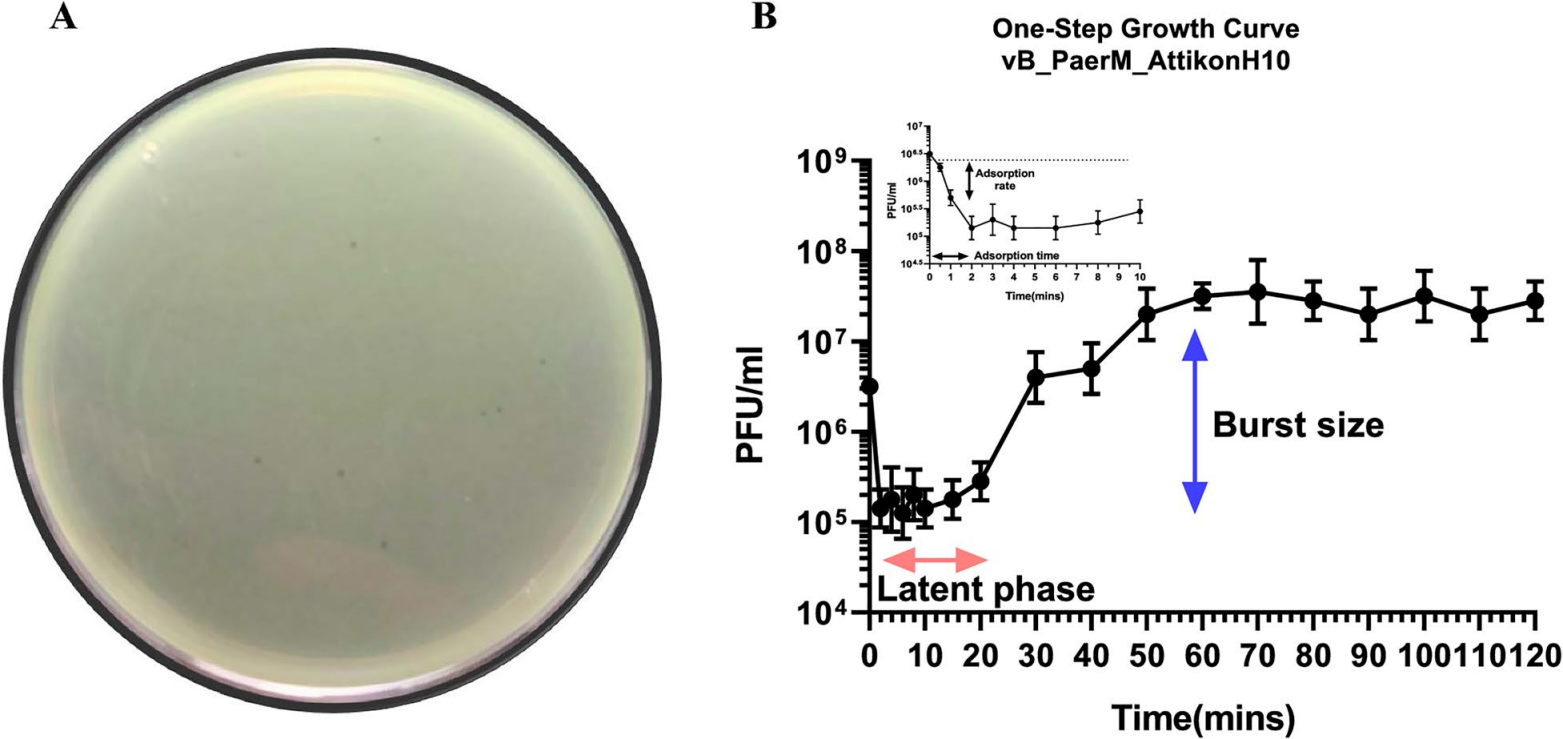
Plaque assay of lytic jumbo phage (vB_PaerM_AttikonH10) isolated from a susceptible *P. aeruginosa* isolate (AUHB77) (**Α**) and its microbiological characteristics, including adsorption rate, adsorption time, latent phase and burst size calculated using one-step phage growth curve (**B**). The data shown represent the mean values from two independent replicates, and the error bars indicate the standard deviations

In an effort to identify new phages that could efficiently infect *P. aeruginosa* multiple samples of wastewaters, stools and rectal swabs were used. Using spot tests, samples were examined for the presence of phages. Plaques with unique morphologies were chosen for further research. There were many phage plaques seen on different *P. aeruginosa* strains. Through multiple rounds of plaque testing, a tiny, transparent plaque with clear lysis and a diameter of around 0.5 mm on 0.5% soft agar was isolated using the susceptible AUHB77 *P. aeruginosa* strain and purified (Fig. 1A). After purification phage titers were ~ 10^10^ PFU/mL. Phage seeds were stored in filtered LB+ medium at 4 °C until further use, with stability monitored monthly.

### Determination of host range

The host range of the jumbo phage AttikonH10 was determined against 30 clinical isolates (4 NDM_,_ 16 VIM, and 10 susceptible to antibiotics) (Table 1). Strong lytic activity with clear or semi-clear lysis was found in 83.3% (25/30) of isolates tested. Remarkably, phage AttikonH10 demonstrated clear or semi-clear lysis throughout the host range and was able to effectively infect 17/20 (85%) of the isolates harboring *bla*_VIM_ and *bla*_NDM_ genes, making it an attractive option against MBL producing *P. aeruginosa* isolates.

**Table 1:** Host range of isolated jumbo phage vB_PaerM_AttikonH10 against 30 *P. aeruginosa* clinical isolates part of Attikon University Hospital collection harboring different resistance mechanisms

Resistance mechanism	Total Isolates (N)	Clear Lysis (+ + +)	Semi-clear Lysis (+ +)	Turbid Lysis ( +)	No Lysis ( −)
None	10	6	2	0	2
VIM	16	8	6	0	2
NDM	4	1	2	1	0
Total	30	15	10	1	4

Plates were examined for the presence of plaques and they were assessed based on the clarity of plaques, which was scored as transparent (+ + +), semi-transparent (+ +), turbid ( +) and no inhibition (-)

### One-step phage growth curve

The one-step growth curve was performed using the clinical isolate AUHB77 as the host strain. The assay was conducted at 37 °C in triplicate, and the results represent the mean values of these independent replicates. By 2 min post-infection, an average of 94% of the initial virions had been adsorbed to the host cells. The latent period was estimated to be approximately 20 min, and the rise phase began shortly after, with complete replication occurring within 60 min. The calculated burst size was approximately 165 PFU per infected cell (Fig. 1B).

### Growth curves of host bacteria

**Fig. 2:**
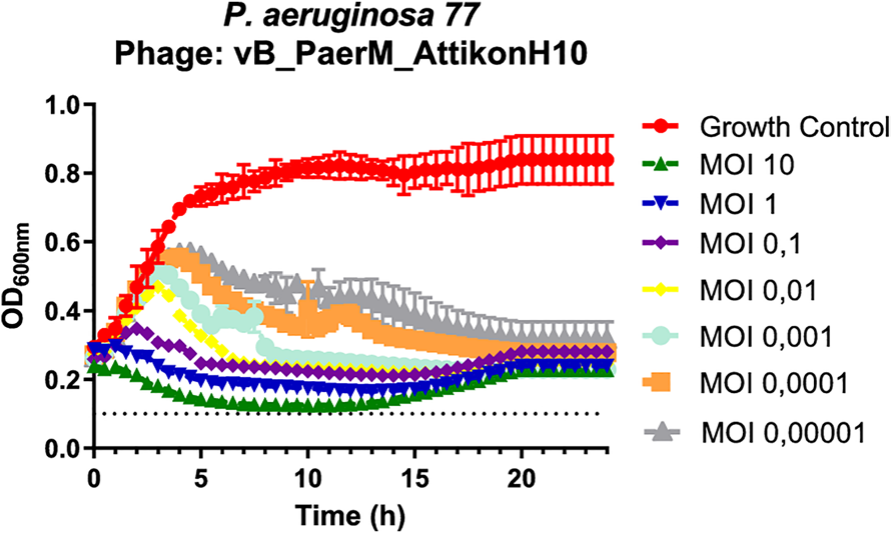
Growth curves of jumbo bacteriophage vB_PaerM_ AttikonH10 against host isolate. The experiment was performed in triplicate and bars indicate standard deviations. Horizontal dotted lines represent the background optical density at 600 nm (OD_600_)

Individual replicates have been tested, and the mean with standard deviation was plotted. After phage addition, OD reduction close to background levels at 6 h was observed. Jumbo phage AttikonH10 exhibited strong lytic activity against the host strains in growth curves with 90–109% growth inhibition even at the lowest MOI (10^–5^) tested (Fig. 2). Growth reduction of 102–109% was found at MOIs ≥ 0.001 indicating that OD at t = 24 h was lower than the OD at t = 0 h i.e. no regrowth was observed.

### Genomic and taxonomic analysis

De novo assembly has resulted in a single (pseudo)circular scaffold of 278,406 bp, implying that the complete non-redundant genome of the phage was acquired, with no defined termini for the respective linear dsDNA molecule discernible from raw read pile up unto it. An automated genome annotation resulted in 413 predicted open reading frames (ORFs), 86 of which had a functional assignment, as well as 6 tRNAs (Supplementary Table 2). None of the annotated ORF products was revealed to be associated with either temperate phage lifestyle, potential increase in the virulence of the host bacterium, or conferment of drug resistance. Intergenomic similarity to the publicly available phage genomes reveals that the bacteriophage AttikonH10 belongs to the class *Caudoviricetes*, family *Chimalliviridae*, genus *Phikzvirus*, and might be considered a newly reported isolate representing *Phikzviru*s *phiKZ* species with whom it shares > 95% intergenomic similarity over the complete genome lengths. Moreover, the presence of the auxiliary metabolic genes (AMGs), such as *PhuZ* (XLY82630) and *ChmA* (XLY82604), which play a crucial role in the formation of a nucleus-like structure during virion progeny, allows us to hypothesize that, besides the high intergenomic similarity, bacteriophage AttikonH10 shares a similar replication strategy. As *Pseudomonas* phage phiKZ itself is one of the representative examples of a “headful” packaging phage, whose genomes are linear dsDNA molecules that are terminally redundant and circularly permuted within a population of progeny virions [44], we have opted to “open” the genome at the start of *TerL* ORF.

**Fig. 3:**
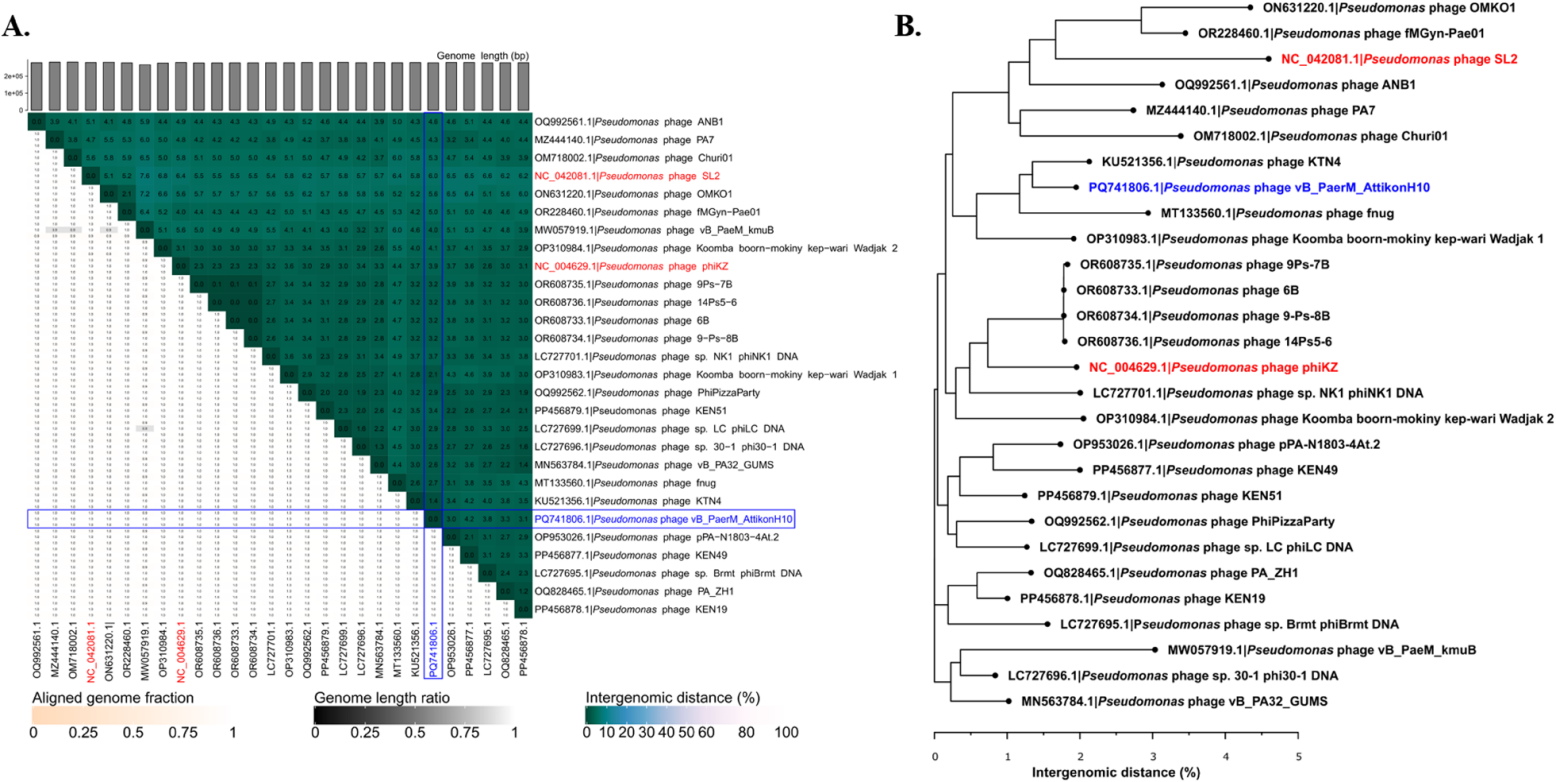
**A** VIRIDIC-generated heatmap showing pairwise intergenomic distances between *Pseudomonas *phage vB_PaerM_AttikonH10 and other recognized or tentative Phikzvirus phage genus representatives that have complete genomes elucidated (n = 1 + 27). Blue rectangles indicate values associated with the studied phage. **Β** VIRIDIC-calculated complete genome nucleotide sequence intergenomic distance Neighbour-Joining (NJ) tree. The NJ tree was drawn from a pairwise intergenomic distance matrix generated by VIRIDIC for a dataset comprising the studied *Pseudomonas *phage vB_PaerM_AttikonH10 and other recognized or tentative Phikzvirus phage genus representatives that have complete genomes elucidated (n = 1 + 27). The tree is unrooted and drawn to scale; the scale bar represents genome nucleotide sequence divergence percentages. Tip labels follow the format of “Genome accession|Phage”. Tip labels of bacteriophages from RefSeq are colored red. Tip label of phage vB_PaerM_AttikonH10 is in blue"

**Fig. 4:**
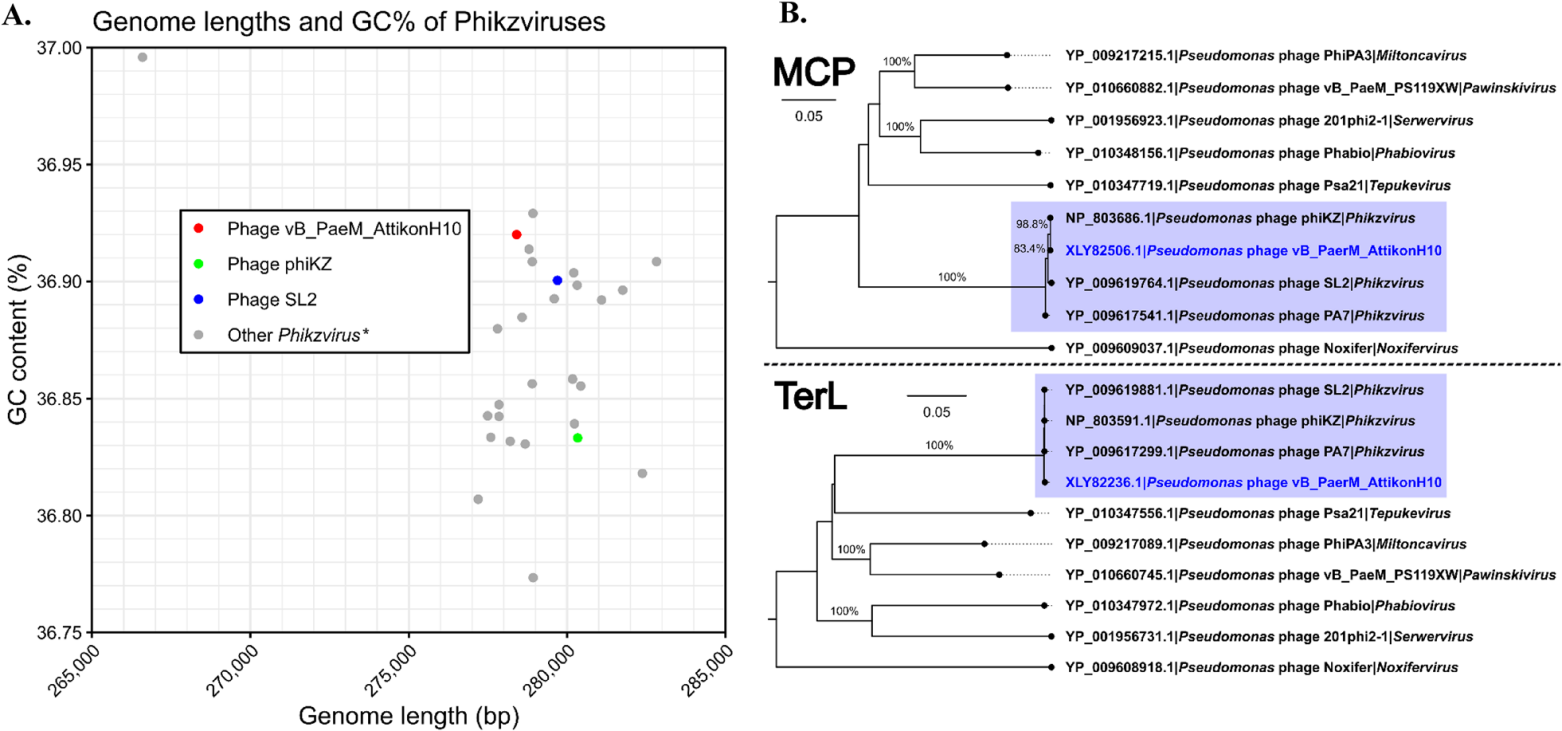
**Α** Genome lengths and GC% of Phikzviruses. Y axis represents GC% content, X axis—genome length (bp). Points represent individual phage complete genomes. Studied Pseudomonas phage vB_PaerM_AttikonH10 and other phages of interest are colored according to the legend. **B** Major Capsid Protein (MCP; top) and Terminase Large subunit (TerL; bottom) phylogenetic trees with the respective aa sequences from vB_PaerM_AttikonH10 within the context of nine highest-scoring hits from other viruses found in RefSeq. The NJ trees from MCP and TerL amino acid sequence multiple sequence alignments representing the datasets comprising the MCP of the studied *Pseudomonas *phage vB_PaerM_AttikonH10 and other Pseudomonas-infecting viruses from RefSeq encoding homologous MCP and TerL (n = 1 + 9 in both cases, respectively) are shown. The trees are midpoint rooted. The trees are drawn to scale with the scale bar representing amino acid substitutions per site. There was a total of 734 and 716 positions in the final MSAs used for generating the MCP and TerL trees, respectively. Branch bootstrap support percentages are shown only for branches supported by at least 80% replicates (out of 1000 replicates). Tip labels follow the format of “Protein accession|Phage|Genus”. Clade comprising *Pseudomonas *phage vB_PaerM_AttikonH10 and other Phikzviruses, including their most-recent common ancestor node, is colored in blue. Tip label of phage vB_PaerM_AttikonH10 is highlighted in blue

**Fig. 5:**
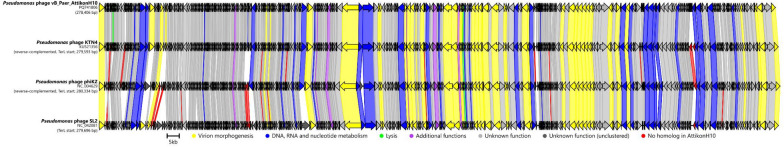
Genome organization and proteome content comparison of *Pseudomonas* phage vB_PaerM_AttikonH10 to its closest relative—phage KTN4, as well as two taxonomically recognized Phikzvirus representatives (SL2 and phiKZ). Genomes are drawn to scale; the scale bar indicates 5000 base pairs. The pseudocircular assembly of the phage vB_PaerM_AttikonH10 genome was artificially opened at the start of TerL gene during the auto-annotation. Genome representations of other phages in the comparison were re-organized as indicated to ensure collinearity with the vB_PaerM_AttikonH10. Arrows representing open reading frames point in the direction of the transcription and are color-coded based on the function of their putative product according to the legend. vB_PaerM_AttikonH10 genome was auto-annotated using the Phanotate and Pharokka combination (original annotations from downloaded GenBank files were retained for other genomes). Ribbons connect phage proteins sharing > 70% amino acid sequence similarity and are colored in lighter shades according to the predicted functional group of the respective phage vB_PaerM_AttikonH10 ORF products (see legend for details)

*Pseudomonas* phage KTN4 was determined to be the most intergenomically similar bacteriophage to AttikonH10, with an intergenomic distance of merely ~ 1% when their complete genome nucleotide sequences are compared (Fig. 3A, B). When GC% and genome lengths of complete known or tentative *Phikzvirus* genomes are compared, AttikonH10 is on the higher end of genome GC% (third largest one out of 28) and on the lower end (20 out of 28) of genome lengths (Supplementary Table 1). However, both these parameters largely show little variation across the representatives of this same phage genus (Fig. 4A). MCP further supported that AttikonH10 is a *Phikzvirus* representative. Furthermore, the MCP of the studied phage demonstrated complete amino acid sequence identity to the counterpart from the genus namesake – *Pseudomonas* phage phiKZ itself, expectedly clustering with other representatives of the genus *Phikzvirus* (Fig. 3B). Similarly, TerL proteins of representative Phikzviruses and AttikonH10 formed a well-defined distinct cluster with little variety within it (Fig. 4B). Such TerL similarity of AttikonH10 to phiKZ further implies the headful packaging strategy to be shared by both. Most of the proteome contents of phage AttikonH10 are homologous to counterparts from other *Phikzvirus* representatives using a 70% protein similarity threshold. However, at least several of the predicted ORFs seem to encode products of a yet unknown function which are not shared with its closest relative or representative *Phikzvirus* genus phages (Fig. 5). As in the case of other Phikzviruses (KTN4, SL2 and phiKZ), no immediately notable particular modular organization of ORFs by their product functional groups is evident. Even when considering only the genes that have their function assigned and that can be categorized as performing similar functions, such coding sequences are frequently interspersed by genes encoding proteins of completely unrelated functions.

### In vivo pharmacokinetics

**Fig. 6:**
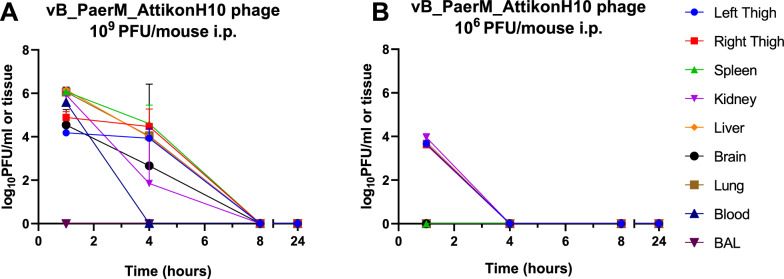
Pharmacokinetics of vB_PaerM_AttikonH10 in vivo via IP route. The phage titre in log_10_ PFU/mL of tissue after 1, 4, 8 and 24 h of phage administration at a dose of 10^9^ PFU/mouse (**A**) and 10^6^ PFU/mouse (**B**). The bars indicate the mean (± SD) from two independent animals

Maximum phage titres were found at 1 h post-phage IP injection for both phage doses of 10^9^ and 10^6^ PFU/mouse, although phage titres were lower using 10^6^ PFU/mouse dosing regimen. For group received 10^9^ PFU/mouse, the median phage titers in well-perfused organs (spleen, lungs, liver, kidneys, and blood) were significantly higher (6.07 ± 0.20 log₁₀ PFU/mL of tissue) compared to lower-access tissues, including the right thigh, left thigh, and brain (5.04 ± 0.72 log₁₀ PFU/mL of tissue, p < 0.01) (Fig. 6A). The reduced accumulation in these sites likely reflects lower vascularization and, in the case of the brain, the restrictive nature of the blood–brain barrier. No phages were found in BAL. For group received 10^6^ PFU/mouse, phage titres at 1 h was only detectable in kidneys, liver, left and right thigh (4 ± 0.23 log_10_ PFU/tissue) (Fig. 6B). After 4 h, there was a gradual decrease in phage titres in all organs and the phages were completely cleared at 8 h and 4 h in groups that received 10^9^ and 10^6^ PFU/mouse, respectively. Mean ± SD elimination half-lives, in group that received 10^9^ PFU/mouse, in different tissues were 3.48 ± 0.17 h.

### In vivo efficacy

**Fig. 7:**
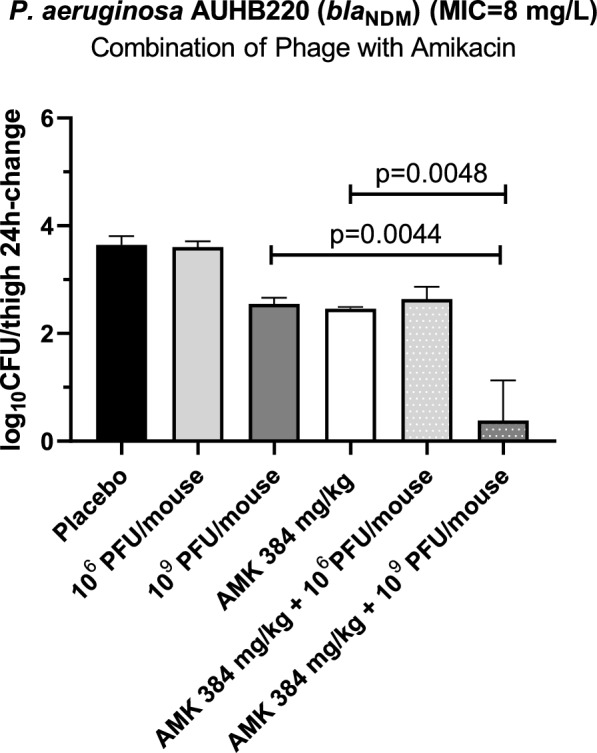
In vivo activity of jumbo phage alone and in combination with amikacin in a thigh infection mouse model. Error bars indicate standard deviations. Bacterial log_10_ cfu/thigh change after 24 h of treatment compared to bacterial load before start of treatment. Significant p values of one-way ANOVA followed by post-test are shown

The average ± SD bacterial load at the start of treatment (e.g. 1 h post-infection) was 5.53 ± 0.11 log_10_ cfu/thigh. In the placebo group, the bacterial burden increased to 9.28 ± 0.15 log_10_ cfu/thigh at 24 h of treatment (e.g. 25 h post-infection), corresponding to an approximate increase of 3.75 log_10_ cfu/thigh from the bacterial burden at start of therapy. Phage therapy at the lowest dose of 10^6^ PFU/mouse did not have an effect. Monotherapy with either jumbo phage at 10^9^ PFU/mouse q12h or amikacin at 384 mg/kg/day resulted in a reduction of bacterial load in thigh tissue compared to the placebo group (1.09 ± 0.11 log_10_ cfu/thigh, p = 0.065 and 1.19 ± 0.03 log_10_ cfu/thigh, p = 0.058, respectively). Notably, the combination of amikacin and phage dosing regimen of 10^9^ PFU/mouse resulted in a greater bacterial reduction compared to the placebo group (2.07 ± 0.74 log_10_ cfu/thigh), which was statistically significant when compared to either monotherapy regimens (p = 0.0044–0.0048) (Fig. 7). No significant effect was found for the combination therapy of amikacin with the lowest dose of AttikonH10 phage at 10^6^ PFU/mouse. In vivo pharmacokinetic data revealed that using 10^6^ PFU/mouse, phage titres in the infected thigh remained low (4 ± 0.23 log_10_ PFU/thigh) at 1 h post-administration. The low phage titers observed at 10^6^ PFU/mouse likely explain the lack of bacterial load reduction in both phage monotherapy and combination therapy groups.

## Discussion

In this study, we report the characterization of the jumbo bacteriophage AttikonH10, a member of the *Phikzvirus* genus within the *Chimalliviridae* family, isolated from sewage water in Athens, Greece. Given the fact that the new bacteriophage was isolated from a biodiversity-rich environment (i.e., sewage), the co-evolution of phage and bacteria could select for phage genomes with richer molecular machineries against the variety of sufficiently distinct susceptible host bacteria found nearby [45]. This form of co-evolution mechanism was projected to bestow many alterations upon the phage, allowing it to infect closely-related bacterial pathogens with different phenotypes [46]. Keeping that in mind, the purification of phages from Greece's central treatment plant, which is currently dealing with a disastrous crisis involving MDR bacteria [47], strongly suggests that this phage was already circulating through the population.

AttikonH10 phage produced small, clear plaques, which is typical for *Pseudomonas* jumbo lytic phages [48]. Biological characterization revealed rapid adsorption kinetics and a short latent period, supporting its suitability for therapeutic use. AttikonH10 exhibited potent lytic activity against a broad range of *Pseudomonas aeruginosa* clinical isolates, including MBL-producing strains. Notably, AttikonH10 infected 85% of tested MBL-producing isolates and demonstrated effective bacterial growth inhibition at clinically relevant phage titres [49].

AttikonH10 shares key features with other *Phikzvirus* phages, including a short latent period and high burst size, supporting its efficient replication. Most *Phikzvirus* phages, including AttikonH10, have been isolated from sewage waters and show strong lytic activity against *P. aeruginosa*, highlighting their therapeutic potential. The *Phikzvirus* phage genus has been relatively recently classified into a new viral family named *Chimalliviridae* [50]. Phages belonging to *Chimalliviridae* family have ability to form a proteinaceous, nucleus-like compartment during infection, primarily composed of chimallin (*ChmA*). It has been proposed that the bacteriophage nucleus structure serves multiple roles, such as protecting phage DNA from host’s molecular “scissors” (various nucleases), organising phage’s replication and translational phases, as well as orchestrating virion assembly [51–53]. These structural and functional complexities make *Phikzvirus* phages promising tools in phage therapy, particularly against MDR isolates. Despite classification into the same species, jumbo phages often harbour numerous non-homologous ORFs unique to individual isolates, with potential effects on host range and therapeutic efficacy. For example, jumbo phages with > 95% intergenomic similarity have been shown to carry up to 24 unique ORFs, indicating substantial genomic variability [54]. In *Phikzvirus* phages, even small intergenomic percentage differences across their large genomes (~ 280 kb) may result in significant phenotypic differences. While many of these unique ORFs encode hypothetical proteins, their biological functions remain to be elucidated. Future comparative analyses of multiple *Phikzvirus* isolates could provide insights into the roles of this genetic variability, particularly in optimizing their use for clinical applications.

In vivo pharmacokinetics was shown that jumbo phage AttikonH10 demonstrated rapid distribution across tissues following intraperitoneal administration, with peak titres observed at 1 h post-phage IP injection, consistent with previous studies on different phage types [55]. Tissue distribution was dose-dependent as dosing regimen of 10^9^ PFU/mouse, reached significantly higher phage titers in well-perfused organs (e.g., spleen, liver, kidneys, lungs, and blood; 6.07 ± 0.20 log_10_ PFU/mL of tissue) than in difficult to reach tissues (e.g., thighs and brain; 5.04 ± 0.72 log_10_ PFU/mL of tissue), likely reflecting reduced perfusion and physical barriers such as the blood–brain barrier. In contrast, at the lower dose of 10^6^ PFU/mouse, phage titres were lower (4 ± 0.23 log_10_ PFU/mL of tissue) and rapidly cleared 4 h post-phage IP injection. The average elimination half-life at 10^9^ PFU/mouse was 3.48 ± 0.17 h, slightly shorter than previously reported for smaller podophages (5.3–7.48 h), possibly due to more efficient in vivo clearance of larger virions (Phikzviruses have ~ 145 nm capsid diameter and ~ 200 nm long tails [56]), which are more susceptible to filtration or immune recognition [14]. The phage–amikacin combination showed considerable in vivo therapeutic efficacy, consistent with previous studies reporting enhanced bacterial clearance through phage–antibiotic synergy [57, 58]. High-dose phage monotherapy (10^9^ PFU/mouse) significantly reduced bacterial burden (1.09 ± 0.11 log_10_ cfu/thigh), comparable to a humanized amikacin regimen (1.19 ± 0.03 log_10_ cfu/thigh), while their combination achieved an even greater reduction (2.07 ± 0.74 log_10_ cfu/thigh) compared to placebo. Importantly, no phage-resistant mutants were detected during treatment, supporting the robustness of AttikonH10 as a therapeutic candidate. In contrast, the 10^6^ PFU/mouse dose, showed no reduction in bacterial load either alone or in combination with amikacin. These results suggest a threshold phage titre is required for efficacy, conceptually analogous to the MIC used in antibiotic therapy. Together, these findings underscore the critical role of dosing and pharmacokinetics in optimizing jumbo phage therapy and highlight the need for defining a minimum effective phage dose for clinical translation.

While our study provides important insights into the therapeutic potential of jumbo phages, several limitations should be acknowledged. First, the dose–response relationship of phage therapy was not comprehensively explored. Assessing a broader range of doses would offer a more detailed understanding of the minimum effective dose and the therapeutic window. Second, although AttikonH10 exhibited genomic features consistent with phiKZ-like jumbo phages, including hallmark structural and transcription-related genes, we did not perform fluorescence microscopy to visualize the formation of the proteinaceous nucleus-like structure typically associated with this phage group. As such, the intracellular organization and infection dynamics of AttikonH10 remain to be confirmed experimentally.

## Conclusions

In conclusion, a *Pseudomonas* jumbo phage AttikonH10 with a strong lytic activity against a wide range of MBL-producing CRPA clinical isolates has been isolated and characterized. AttikonH10 is a strictly lytic representative of the *Phikzvirus* genus, and is deemed genomically safe for potential therapeutic applications. Coupled with a high adsorption rate, short latent period, and a good burst size, features of AttikonH10 make it a good candidate for phage therapy. Moreover, our study demonstrated the therapeutic efficacy of jumbo phage AttikonH10 using dosing regimen of 10^9^ PFU/mouse, to be similar to amikacin, showing a greater effect when combined together, making it a viable substitute therapy against CRPA clinical isolates.

## Supplementary Information

Additional file 1.

Additional file 2.

## Data Availability

The complete functionally annotated genome of *Pseudomonas* bacteriophage vB_PaerM_AttikonH10 is publicly available from the GenBank database under the following accession number: PQ741806.1. Other data are available upon request
