# Publisher Correction: Therapeutic application of a jumbo bacteriophage against metallo-β-lactamase producing *Pseudomonas aeruginosa* clinical isolates

**DOI:** 10.1186/s12929-025-01200-3

**Published:** 2025-11-24

**Authors:** Paschalis Paranos, Dimitrios Skliros, Nikita Zrelovs, Panagiota‑Christina Georgiou, Karina Svanberga, Andris Kazaks, Marios Kostakis, Nikolaos Thomaidis, Emmanouil Flemetakis, Joseph Meletiadis

**Affiliations:** 1 Clinical Microbiology Laboratory, Medical School, Attikon University Hospital, National and KapodistrianUniversity of Athens, Rimini 1, Haidari, Athens, Greece; 2 Laboratory of Environmental Biotechnology, Department of Biotechnology, School of Applied Biology and Biotechnology, Agricultural University of Athens, Athens, Greece; 3 Latvian Biomedical Research and Study Centre, Riga, Latvia; 4 Laboratory of Analytical Chemistry, Department of Chemistry, National and Kapodistrian University of Athens, Athens, Greece

**Publisher Correction: Journal of Biomedical Science (2025) 32:74** 10.1186/s12929-025-01169-z

After publication of the article, it was brought to our attention that Fig. 1 and Fig. 2 are misplaced and should be swapped. The correct figure captions of the Figs. 1 and 2 are shown below:

**Fig. 1:**
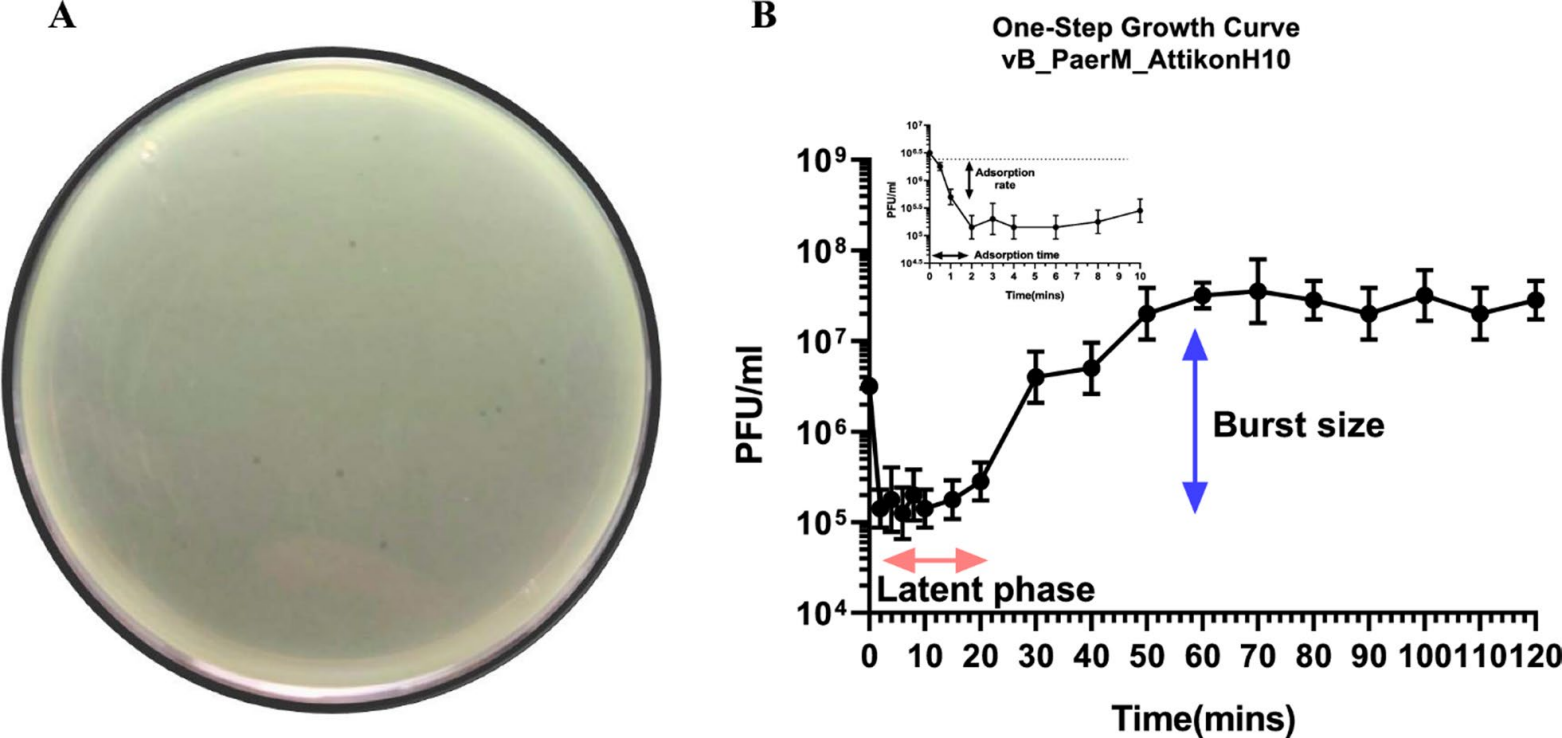
Plaque assay of lytic jumbo phage (vB_PaerM_AttikonH10) isolated from a susceptible *P. aeruginosa* isolate (AUHB77) (**Α**) and its microbiological characteristics, including adsorption rate, adsorption time, latent phase and burst size calculated using one-step phage growth curve (**B**). The data shown represent the mean values from two independent replicates, and the error bars indicate the standard deviations

**Fig. 2:**
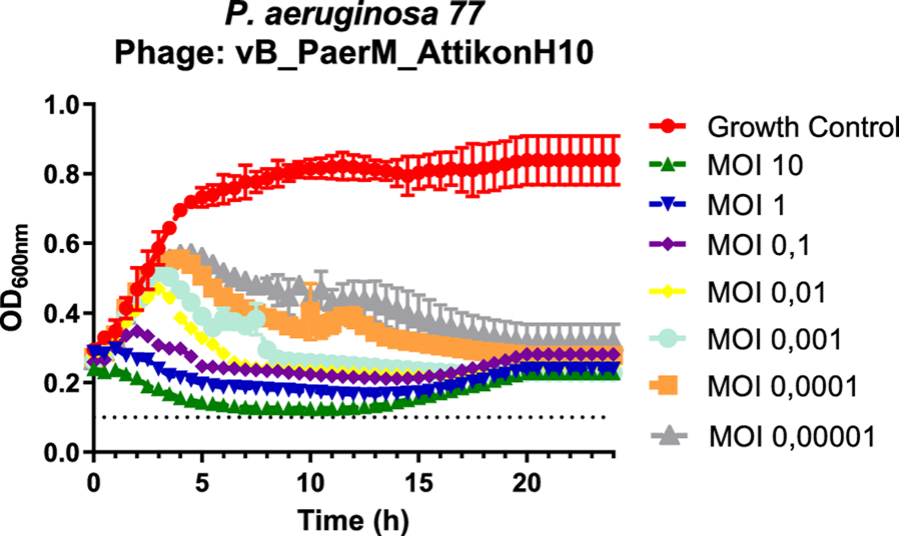
Growth curves of jumbo bacteriophage vB_PaerM_ AttikonH10 against host isolate. The experiment was performed in triplicate and bars indicate standard deviations. Horizontal dotted lines represent the background optical density at 600 nm (OD600)

The original publication [1] has been updated. The publisher apologizes to the authors and readers for the inconvenience caused.
